# Predictors for identifying autoimmune encephalitis in pediatric patients

**DOI:** 10.3389/fcimb.2026.1827367

**Published:** 2026-07-02

**Authors:** Yanmeng Sun, Shangmin Yang, Mengyuan Wang, Huan Xu, Shifu Wang

**Affiliations:** 1 Department of Microbiology Laboratory, Children’s Hospital Affiliated to Shandong University (Jinan Children’s Hospital), Jinan, China; 2 Department of Clinical Microbiology, Shandong Provincial Clinical Research Center for Children’s Health and Disease, Jinan, China; 3 Department of Scientific Affairs, Vision Medicals Center for Infectious Diseases, Guangzhou, China

**Keywords:** autoimmune encephalitis, metagenomic next-generation sequencing, pediatrics, predictors, ROC curve

## Abstract

**Objectives:**

This study aimed to identify the independent predictors and develop a predictive model for autoimmune encephalitis (AE) in pediatric populations.

**Methods:**

This retrospective study comprised 88 pediatric patients with encephalitis (37 AE cases and 51 non- AE cases) at Children’s Hospital Affiliated to Shandong University between May 2020 and April 2025. Lasso regression analysis, univariate and multivariate logistic analysis was used to identify autoimmune encephalitis associated risk factors. The nomogram visualized the results. Receiver operating characteristic (ROC) curves, calibration plots, Brier scoring and decision curve analysis (DCA) were used to evaluate the diagnostic model.

**Results:**

16 clinical variables significantly differed between the autoimmune encephalitis and non-autoimmune encephalitis groups. Lasso regression analysis, univariate and multivariate logistic analysis identified four significant independent predictors: age (OR: 1.44; 95% CI: 1.09–1.91; *P* = 0.010), proteins in the cerebrospinal fluid/100(C.Protein.100) (OR: 0.80; 95% CI: 0.65–1.00; *P* = 0.049), chloride in the cerebrospinal fluid(C. Chloride) (OR: 1.38; 95% CI: 1.00–1.92; *P* = 0.050), and spontaneous remission (OR: 21.14; 95% CI: 3.17–141.17; *P* = 0.002) were risk factors for autoimmune encephalitis. The predictive model demonstrated excellent discrimination (AUC 0.976, 95% CI 0.947-1.000) and calibration (Hosmer-Lemeshow *p* = 0.886, R²=0.9796, Brier score 0.052).

**Conclusions:**

This study established and validated a high-performance predictive model incorporating four clinically accessible parameters for the diagnosis of pediatric autoimmune encephalitis.

## 1 Introduction

Encephalitis is a severe inflammatory disorder of the brain with many possible causes and a complex differential diagnosis. Existing criteria for autoimmune encephalitis are too reliant on antibody testing and response to immunotherapy, which might delay the diagnosis (Graus et al., 2016). However, not all the antibodies of patients with autoimmune encephalitis (AE) are positive (Dalmau and Graus, 2023). Some patients with autoimmune encephalitis might not respond to immunotherapy, Conversely, patients with other disorders might respond to immunotherapy (Titulaer et al., 2013). The estimated incidence of encephalitis in high-income countries is about 5–10 per 100,000 inhabitants per year; encephalitis affects patients of all ages and represents a significant burden to patients, families, and society (Jmor et al., 2008; Vora et al., 2014). AE accounts for approximately 10% to 20% of all encephalitis cases (Ren et al., 2021). One of the studies from the Netherlands showed that from 2015 to 2018, the autoimmune encephalitis incidence rate was 1.54 per million children per year (de Bruijn et al., 2020). Three additional studies (from the United Kingdom, Denmark, and Hong Kong, China) also reported the incidence rates of NMDAR - AE in the child population, which were 0.85 cases, 4.2 cases, and 2.2 cases per million children per year, respectively (Wright et al., 2015; Ho et al., 2018; Boesen et al., 2019).

The diagnosis of AE in pediatric populations remains clinically challenging due to four challenges: (1) the clinical manifestations (e.g., abnormal mental behavior, epilepsy, and memory impairment) are more insidious and less typical. (2) communication and assessment are difficult. (3) the range of differential diagnoses is broader, and severe infections are more common. (4) interpretation and limitations of auxiliary examinations. Consequently, these diagnostic constraints highlight the urgent need for the identification of pediatric-specific predictors and the establishment of predictive models for pediatric AE to improve early detection and clinical outcomes.

In recent years, research on risk factors for AE in pediatric populations has gained increasing attention. Among the most significant and well-studied risk factors, specific antibody types are highly correlated with specific tumors (Lancaster et al., 2010; Titulaer et al., 2013; Graus et al., 2016). A history of herpes simplex virus (HSV) encephalitis is an established risk factor for the development of secondary autoimmune encephalitis, particularly anti-N-methyl-D-aspartate receptor (NMDAR) encephalitis, and other antibody-related encephalitis as well (Armangue et al., 2014). Age and gender are also considered as risk factor (Graus et al., 2016). Genetic susceptibility is currently a leading research area (Bien et al., 2012; Kim et al., 2017). Despite these insights, current research remains disproportionately focused on adult populations, resulting in critical knowledge gaps regarding pediatric-specific risk stratification, validated biomarker thresholds, and multifactorial pathogenic interactions. These limitations significantly hinder the development of precision diagnostic and therapeutic frameworks for AE in children. Thus, we performed a comprehensive analysis of AE predictors in pediatric patients through a retrospective case-control study encompassing 88 cases of encephalitis (37 AE cases vs 51 controls) in this study.

## 2 Materials and methods

### 2.1 Study design and participants

The diagnostic criteria of AE followed by the expert group are mainly based on international diagnostic criteria, include “A clinical approach to diagnosis of autoimmune encephalitis” which published by Lancet Neurol in 2016 (Graus et al., 2016), and “Chinese expert consensus on the diagnosis and management of autoimmune encephalitis (2022 edition)”. Among them, the positive results for anti-neuronal cell antibodies is used as the criterion for diagnosis as proven AE, and the combination of clinical feature, auxiliary examination, reasonable exclusion of alternative causes is used as the criterion of the clinical diagnostic criteria as probable AE.

A total of 88 cases of encephalitis were retrospectively enrolled between May 2020 and April 2025 at the Affiliated Children’s Hospital of Shandong University, among which 37 was AE. Patients enrolled in this study should meet the following inclusive criteria: (1) age<18; (2) diagnosed with encephalitis meeting the consensus diagnostic criteria issued by the International Encephalitis Alliance (2013 revision) (Venkatesan et al., 2013); (3) obtaining cerebrospinal fluid species; (4) having metagenomic next - generation sequencing (mNGS) results. The diagnostic criteria for the AE were as follows (Graus et al., 2016): (1) Subacute onset (rapid progression of less than 3 months) of working memory deficits (short-term memory loss), altered mental status*, or psychiatric symptoms (2) At least one of the following: New focal CNS findings, Seizures not explained by a previously known seizure disorder, cerebrospinal fluid (CSF, abbreviated as C) pleocytosis (white blood cell count of more than five cells per mm³), MRI features suggestive of encephalitis (3) The positive results for anti-neuronal cell antibodies (4) Reasonable exclusion of alternative causes. The clinical diagnosis for both AE and non-AE cases was made by two senior neurologists, based on clinical symptoms, laboratory test results, MRI features, mNGS etiology, and clinical responses to treatment. Clinical data—including sex, age, clinical presentations, MRI features, mNGS tests, and laboratory results—were collected from the patients’ medical histories.

This study was approved by the Ethics Committee of the Children’s Hospital Affiliated to Shandong University (No.: SDFEEB/P-2022017) and conducted in accordance with the Declaration of Helsinki.

### 2.2 Statistical analysis

The data were analyzed using R software (version 4.4.1). Continuous variables were expressed as median (interquartile range, IQR) or mean (standard deviation, SD), and categorical variables as frequencies (%). Group comparisons employed Mann-Whitney U tests (non-normal data) or Student’s *t*-tests (normal data) for continuous variables, and chi-square or Fisher’s exact tests for categorical variables. All available clinical data at the time of admission were systematically collected as candidate variables, encompassing demographic characteristics, clinical manifestations, laboratory parameters, cerebrospinal fluid findings, and neuroimaging features. Prior to variable selection, clinical indicators with a missing rate exceeding 30% were excluded from further analysis to ensure data reliability. For the remaining variables, missing data were handled using K-Nearest Neighbor (KNN) imputation implemented via the VIM package (version 6.2.2) in R with default parameters (k = 5 nearest neighbors), whereby each missing value was replaced by the weighted mean of the corresponding values from the five most similar complete cases, as determined by Euclidean distance across all available variables. All candidate variables were subsequently compared between groups; those achieving statistical significance (p < 0.05) were retained for subsequent LASSO regression analysis. LASSO regression was performed using the glmnet package (version 4.1.10) with 10-fold cross-validation, and the optimal penalty parameter lambda was selected using the minimum cross-validation deviance criterion (lambda. min). Variables with non-zero coefficients at the optimal lambda were entered into multivariable logistic regression for final model development.

### 2.3 Diagnostic model development

Lasso coefficient path plots and cross-validation curves were used to identify predictor significantly associated with autoimmune encephalitis. The final diagnostic model was constructed through sequential univariable and multivariable logistic regression analyses incorporating LASSO-selected predictors. And the forest plots were generated using the R forestploter package (version 1.1.2). To facilitate individualized diagnostic probability estimation, a Nomogram was developed using the rms package (version 8.0.0).

Model discrimination was evaluated using the area under the receiver operating characteristic curve (AUROC) by pROC package (version 1.19.0.1) (Nahm, 2022), and the optimal diagnostic threshold was determined by maximizing the Youden index (sensitivity + specificity − 1). Model calibration was assessed through the Hosmer-Lemeshow goodness-of-fit test and the Brier score using Resource Selection package (version 0.3.6) and Desc Tools package (version 0.99.60), respectively (Zhang et al., 2024). Clinical utility was further quantified by decision curve analysis (DCA) using rmda package (version 1.6), which estimates the net benefit of Nomogram-guided clinical decision-making relative to the default strategies of treating all or no patients across a range of clinically plausible threshold probabilities.

## 3 Results

### 3.1 Patient characteristics

The study enrolled 88 pediatric patients with encephalitis, including 37 AE (42.0%) and 51 non-AE. The cohort consisted of 50 male patients (56.8%), with a median age of 4 years old (interquartile range [IQR] 0.17–8). As shown in **Table 1**, 16 clinical variables significantly differed between the AE and non-AE groups. Biochemical analysis indicated: B.WBC (7.77 vs. 11.16 10^9^/L; *P* = 0.007), B.CRP (0.74 vs. 17.10 mg/L, *P* < 0.001),B.ESR (13.00 vs. 26.00 mm/h; *P* = 0.006), C Cell Count (0.01 vs. 0.14 10^9^/L; *P* < 0.001),C Lym Percent (90.00 vs. 60.00; *P* = 0.002), C Neu Percent (10.00 vs. 40.00; *P* = 0.002), C. Protein (271 vs. 1099 mg/L; *P* < 0.001), C Glucose (3.57 ± 0.53 vs. 2.36± 1.34 mmol/L; *P* < 0.001), C. Chloride (121.89 ± 3.03 vs. 117.03 ± 4.35 mmol/L; *P* < 0.001) in autoimmune encephalitis group. The autoimmune encephalitis group cohort showed higher headache rate (51.4% vs. 9.8%; *P* < 0.001), spontaneous remission rate (75.7% vs. 9.8%; *P* < 0.001), consciousness disorder rate (64.9% vs. 15.7%; *P* < 0.001, seizure rate (54.1% vs. 13.7%; *P* < 0.001) and age (7.00 vs. 0.58; *P* < 0.001). The autoimmune encephalitis group cohort showed longer LOS (24.50 vs. 19.00 P < 0.001) and lower CSF space widening rate (29.7% vs. 58.8% *P* = 0.013).

**Table 1:** Patient’ characteristics, laboratory findings and MRI of AE and non-AE pediatric patients.

Variants	Total (n=88)	non-AE (n=51)	AE(n=37)	P value
Baseline factors				
Age, median (IQR) (year)	4.00 (0.17 to 8.00)	0.58 (0.08 to 3.50)	7.00 (5.00 to 9.00)	<0.001
Gender (male), n (%)	50 (56.8%)	31 (60.8%)	19 (51.4%)	0.507
Clinical manifestations				
Fever, n (%)	74 (84.1%)	43 (84.3%)	31 (83.8%)	1.000
Bloodstream Infection, n (%)	11 (12.5%)	9 (17.6%)	2 (5.4%)	0.11
Upper Respiratory Infection, n (%)	26 (29.5%)	19 (37.3%)	7 (18.9%)	0.104
Pneumonia, n (%)	15 (17%)	12 (23.5%)	3 (8.1%)	0.107
Dizziness, n (%)	6 (6.8%)	1 (2%)	5 (13.5%)	0.079
Headache, n (%)	24 (27.3%)	5 (9.8%)	19 (51.4%)	<.001
Somnolence, n (%)	6 (6.8%)	1 (2%)	5 (13.5%)	0.079
Spontaneous remission, n (%)	33 (37.5%)	5 (9.8%)	28 (75.7%)	<0.001
Consciousness disorder, n (%)	32 (36.4%)	8 (15.7%)	24 (64.9%)	<0.001
Critically. Ill, n (%)	14 (15.9%)	11 (21.6%)	3 (8.1%)	0.159
Convulsion, n (%)	7 (8%)	4 (7.8%)	3 (8.1%)	1.000
Epilepsy, n (%)	3 (3.4%)	1 (2%)	2 (5.4%)	0.57
Seizure, n (%)	27 (30.7%)	7 (13.7%)	20 (54.1%)	<0.001
Reflexes symmetric, n (%)	86 (97.7%)	51 (100%)	35 (94.6%)	0.174
Babinski positive, n (%)	15 (17%)	8 (15.7%)	7 (18.9%)	0.912
Kernig positive, n (%)	3 (3.4%)	2 (3.9%)	1 (2.7%)	1.000
Brudzinski positive, n (%)	6 (6.8%)	5 (9.8%)	1 (2.7%)	0.394
Vomiting, n (%)	29 (33%)	18 (35.3%)	11 (29.7%)	0.750
Diarrhea, n (%)	2 (2.3%)	2 (3.9%)	0 (0%)	0.507
Underlying diseases				
Immunocompromised, n (%)	2 (2.3%)	2 (3.9%)	0 (0%)	0.507
Congenital. Disease, n (%)	3 (3.4%)	3 (5.9%)	0 (0%)	0.261
Laboratory findings				
B.WBC, median (IQR)10^9^/L	10.36 (6.46 to 14.01)	11.16 (7.86 to 15.63)	7.77 (5.71 to 12.54)	0.007
B Neu Percent, median (IQR)	64.20 (51.05 to 76.90)	62.80 (42.40 to 76.75)	69.40 (55.10 to 76.60)	0.184
B Lym Percent, median (IQR)	25.60 (16.25 to 41.40)	29.10 (16.95 to 45.45)	22.80 (16.00 to 35.80)	0.25
B.CRP, median (IQR)(mg/L)	3.68 (0.50 to 23.85)	17.10 (3.50 to 34.30)	0.74 (0.50 to 3.20)	<0.001
B.ESR, median (IQR)(mm/h)	20.00 (11.00 to 37.00)	26.00 (13.00 to 49.00)	13.00 (8.00 to 28.00)	0.006
C Cell Count, median (IQR)10^9^/L	0.04 (0.01 to 0.26)	0.14 (0.02 to 2.56)	0.01 (0.00 to 0.04)	<0.001
C Lym Percent, median (IQR)	87.50 (36.00 to 100.00)	60.00 (25.00 to 92.35)	90.00 (84.20 to 100.00)	0.002
C Neu Percent, median (IQR)	12.50 (0.00 to 64.00)	40.00 (7.20 to 75.00)	10.00 (0.00 to 15.80)	0.002
C Protein, median (IQR)(mg/L)	572.50 (253.50 to 1504.00)	1099.00 (650.50 to 1971.50)	271.00 (221.00 to 336.00)	<0.001
C Glucose, Mean ± SD (mmol/L)	2.87 ± 1.23	2.36 ± 1.34	3.57 ± 0.53	<0.001
C Chloride, Mean ± SD (mmol/L)	119.08 ± 4.53	117.03 ± 4.35	121.89 ± 3.03	<0.001
Magnetic Resonance Imaging				
CSF space widening, n (%)	41 (46.6%)	30 (58.8%)	11 (29.7%)	0.013
Abnormal brain density, n (%)	44 (50%)	25 (49%)	19 (51.4%)	0.109
Ventricular. Dilatation, n (%)	12 (13.6%)	10 (19.6%)	2 (5.4%)	0.109
Outcome				
LOS, median (IQR)	24.50 (16.50 to 34.50)	29.00 (21.00 to 39.50)	19.00 (14.00 to 26.00)	<0.001
Condition improved, n (%)	80 (90.9%)	48 (94.1%)	32 (86.5%)	0.273

B.WBC, the white blood cell count in the blood; B Neu Percent, the percentage of neutrophils in the blood; B Lym Percent, the percentage of lymphocytes in the blood; B.CRP, C-reactive protein in the blood; B.ESR, erythrocyte sedimentation rate in the blood; C Cell Count, the white blood cell count in the cerebrospinal fluid; C Lym Percent, the percentage of lymphocytes in the cerebrospinal fluid; C Neu Percent, the percentage of neutrophils in the cerebrospinal fluid; C Protein, the protein content in the cerebrospinal fluid; C Glucose, the glucose content in the cerebrospinal fluid; C chloride, the chloride content in the cerebrospinal fluid LOS, length of stay.

### 3.2 Selection of predictors and development of nomogram

**Figure 1:**
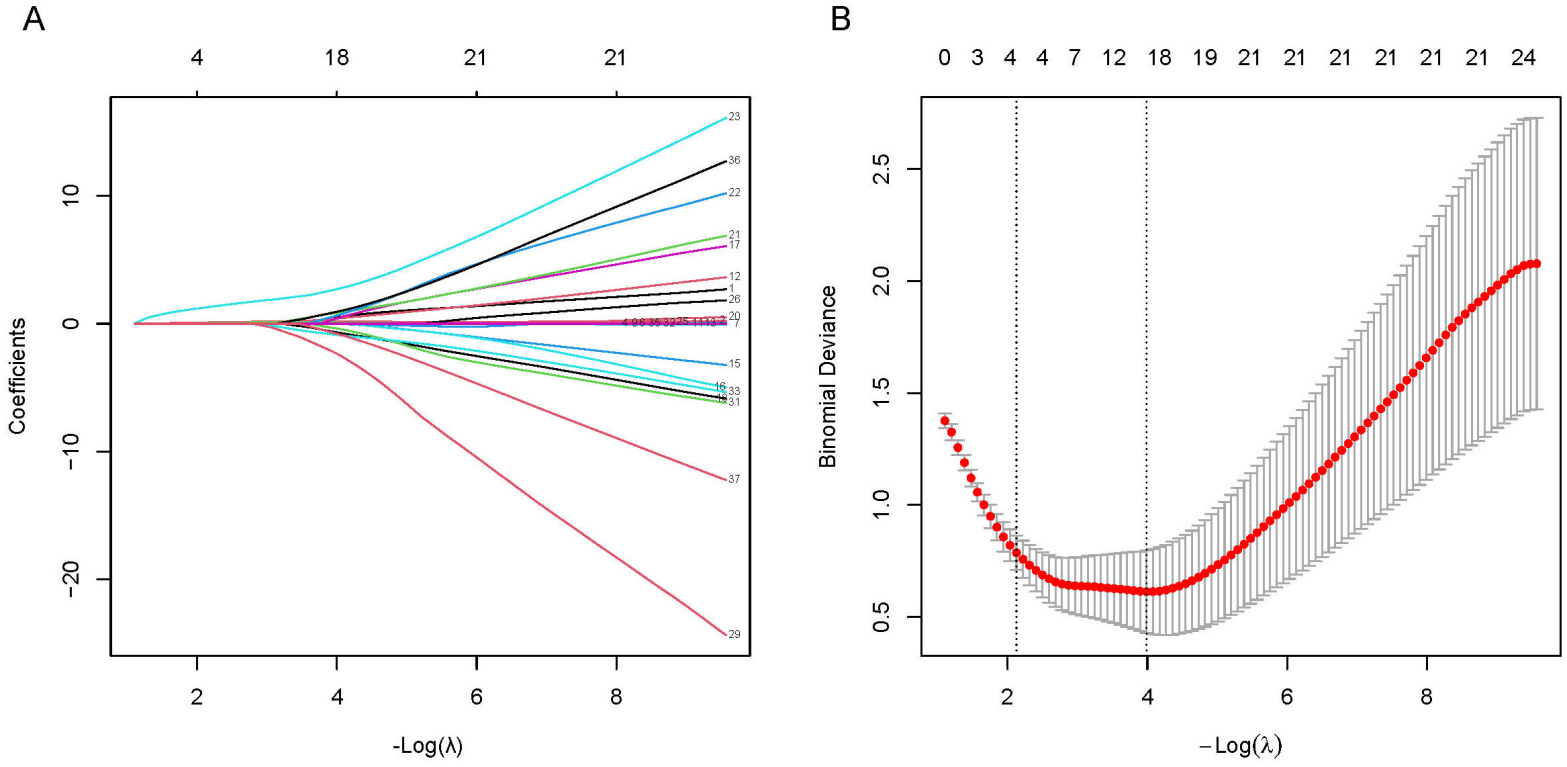
Lasso coefficient path plots **(A)** and cross-validation curves **(B)**.

39 variables between two groups were subjected to LASSO regression. Based on the lambda.1se threshold, 4 variables with non-zero coefficients were ultimately retained as significant predictors. The four features were age, C protein, C chloride, and self-remission (**Figure 1**). Their respective regression coefficients were 0.096, -0.0003, 0.048, and 1.278.

**Figure 2:**
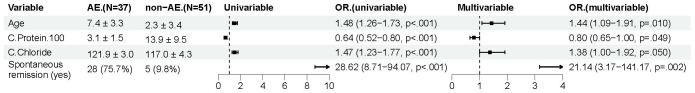
Forest map of 4 risk factors identified by the univariate logistic analysis and multiple logistic analysis for the AE group. C. chloride, the chloride content in the cerebrospinal fluid; C. protein.100, the protein content in cerebrospinal fluid/100.

**Figure 3:**
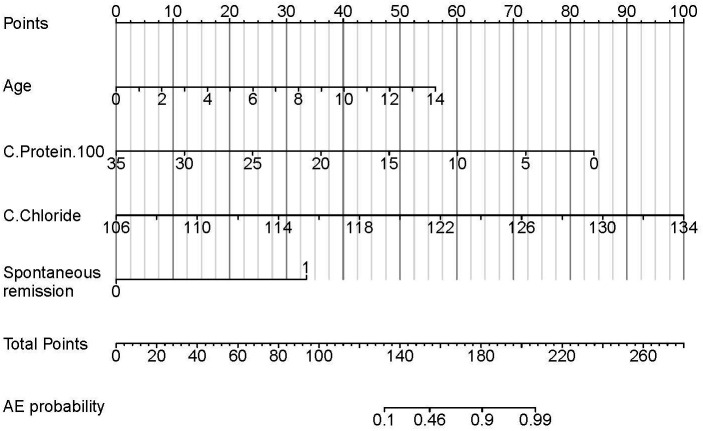
Nomogram for predicting the risk of AE in the pediatric population (including age, C. protein, C. chloride, and spontaneous remission). C. chloride, the chloride content in the cerebrospinal fluid; C. protein.100, the protein content in cerebrospinal fluid/100.To use the nomogram, find the position of each variable on the corresponding axis, and draw a line to the points axis (top) for the number of points. Then, sum up the points from all of the variables, and draw a line from the total points axis to the lower line of the nomogram to determine the predicted risk of AE in the pediatric population.

Multivariable logistic regression analysis with backward stepwise elimination was performed to further analyze the variables. To enhance clinical interpretability, CSF protein levels were rescaled by dividing by 100 before being entered into the multivariable logistic regression model. Thus, the odds ratio (OR) for CSF protein represents the change in risk associated with a 100-unit increase in the original value. After multivariable adjustment, CSF protein (per 0.1mg/L increase) was found as an independent protective factor for AE (OR: 0.80; 95% CI: 0.65–1.00; *P* = 0.049) (**Figure 2**). In contrast, age (OR: 1.44; 95% CI: 1.09–1.91; *P* = 0.010), C. Chloride (OR: 1.38; 95% CI: 1.00–1.92; *P* = 0.050), and self. remission (OR: 21.14; 95% CI: 3.17–141.17; *P* = 0.002) were predictors (**Figure 2**). Thereafter, a prediction model that incorporated the above four independent predictors was developed and presented as a nomogram (**Figure 3**).

### 3.3 The performance of nomogram

**Figure 4:**
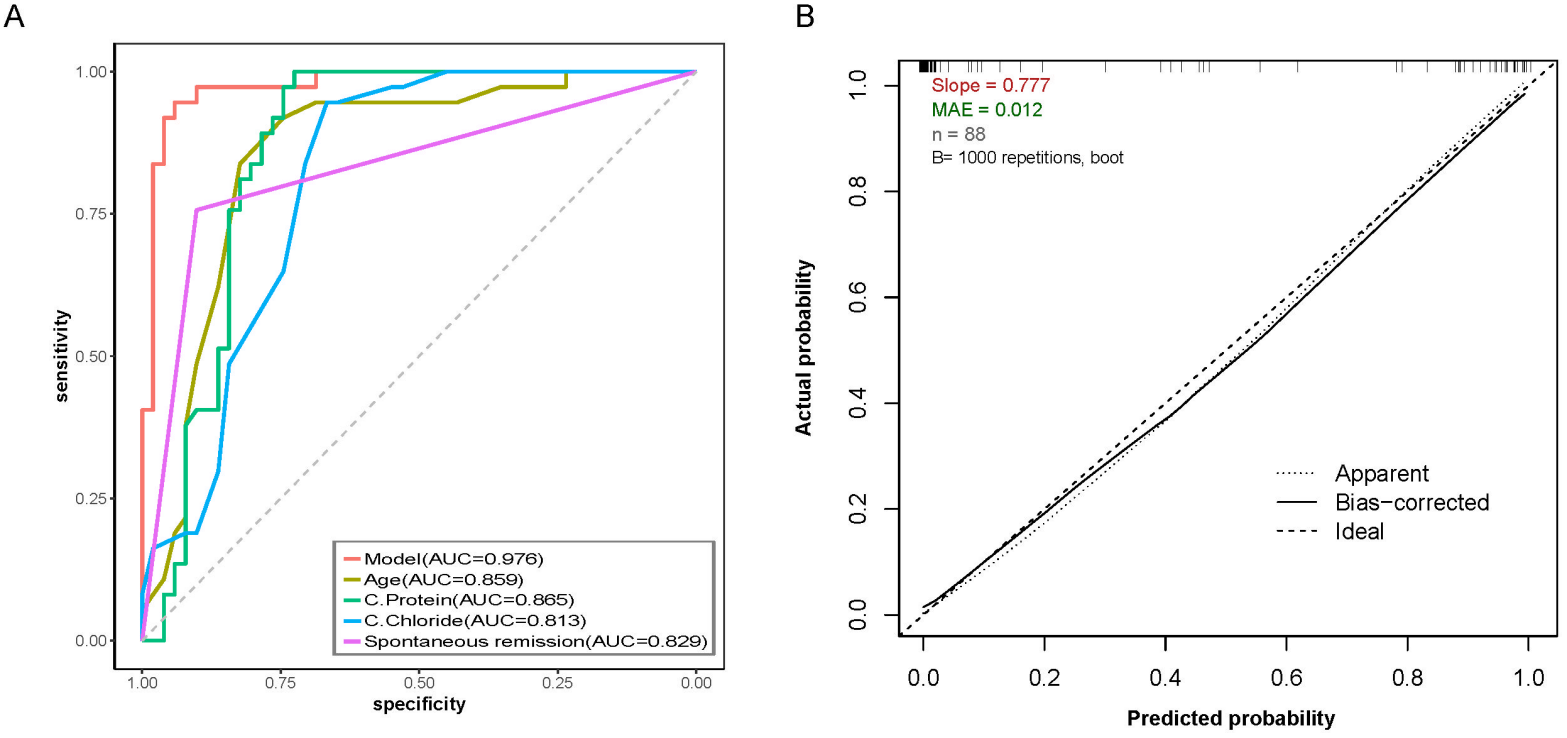
Validation of the model for predicting autoimmune encephalitis (AE) probability. **(A)** The receiver operating characteristic (ROC) curve analysis demonstrated the following area under the curve (AUC) values: model, age, **C** protein, spontaneous remission. **(B)** The calibration plot demonstrated a strong agreement between the predicted probability of autoimmune encephalitis (AE) and the actual observed outcome. C chloride, the chloride content in the cerebrospinal fluid; C protein.100, the protein content in cerebrospinal fluid/100.

The predictive model demonstrated excellent discrimination, with an AUC of 0.976 (**Figure 4A**). Model was satisfactory, as evidenced by: a non-significant Hosmer-Lemeshow test (*P* = 0.886), Calibration was assessed using a bootstrap-corrected calibration curve (1,000 replicates). The mean absolute error (MAE) was 0.012, indicating that the average discrepancy between predicted and observed probabilities was only 1.2%, reflecting clinically acceptable calibration accuracy across the full risk range. The bias-corrected calibration slope was 0.777, deviating from the ideal value of 1.0, suggesting mild overconfidence in extreme predictions—a pattern consistent with the limited sample size and the risk of overfitting indicated by the pmsampsize analysis. Together, these metrics are not contradictory; the low MAE supports overall prediction accuracy, while the slope <1.0 highlights modest over-optimism, a common finding in small-sample logistic models (**Figure 4B**).

### 3.4 The DCA to evaluate its practical application value in clinical decision-making for AE

**Figure 5:**
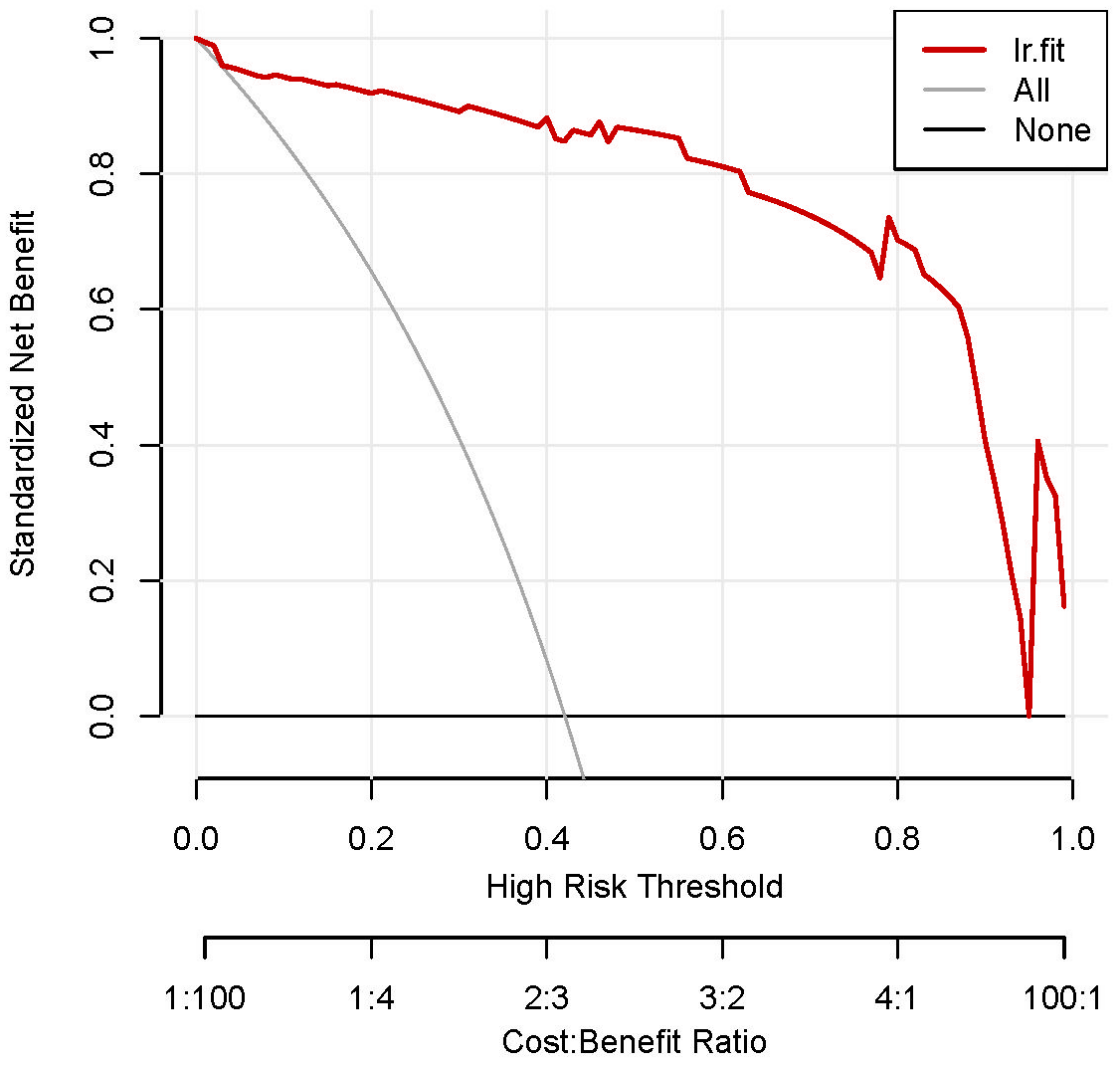
Decision curve analysis of the diagnostic model for AE.

We constructed a decision curve analysis (DCA) to evaluate its practical application value in clinical decision-making (**Figure 5**). The decision curve analysis reveals that the prediction model yields positive net benefits across the entire threshold range (0–0.99) and consistently outperforms the “no - treatment for all” strategy. In the low - risk threshold interval (< 0.42), the net benefit of the model is higher than that of the “treatment for all” strategy, indicating its advantage in avoiding unnecessary interventions. Even at higher thresholds (≥ 0.42), when the net benefit of the “treatment for all” strategy turns negative, the model can still maintain a positive net benefit, suggesting its robust ability to identify high - risk populations. In addition, at extremely high thresholds (> 0.96), the net benefit rebounds again, indicating that the model still has good recognition ability for a very small number of patients at extremely high risk. In conclusion, this model has practical value in clinical decision-making, especially in scenarios where the pros and cons of interventions need to be weighed.

### 3.5 Performance of the model with the cutoff of 0.466 in the total patients

At the optimal cutoff probability of 0.466, the nomogram demonstrated excellent discriminatory performance in the total patient cohort (**Table 2**): sensitivity 0.941 (95% CI: 0.84–0.99), specificity 0.946 (95% CI: 0.82–0.99), AUC 0.976 (95% CI: 0.947–1), and Youden index 0.887. The continuous Net Reclassification Improvement (NRI) comparing the full nomogram (age, CSF protein, CSF chloride, spontaneous remission) with age alone showed significant improvement (overall NRI = 1.480, 95% CI: 1.182–1.760). Among AE patients, 83.8% had appropriately higher probabilities (NRI+ = 0.676, 95% CI: 0.429–0.901); among non-AE patients, 90.2% had appropriately lower probabilities (NRI− = 0.804, 95% CI: 0.628–0.959), confirming that the full nomogram substantially improves risk reclassification over age alone and supports its clinical utility.

**Table 2:** Performance of the model with the cutoff of 0.466 in the total patients.

Prediction	AE	
	1	0
1	35	3
0	2	48
Sensitivity	0.941 (95% CI: 0.84, 0.99)	
Specificity	0.946 (95% CI: 0.82, 0.99)	
AUC	0.976 (95% CI: 0.947-1)	
Overall NRI	1.480 (95% CI: 1.182–1.760)	

## 4 Discussion

Autoimmune encephalitis is a debilitating and potentially treatable condition, more prevalent than infectious encephalitis, starting immunotherapy as early as possible remains an important prognostic factor (Dubey et al., 2018; Broadley et al., 2019; Irani, 2024). This study identified 4 independent risk factors for pediatric AE—age, C. protein, C. chloride, and spontaneous remission—and established a predictive model with an AUC of 0.976. In this multicenter study, we designed and developed an easy-to-use visual nomogram to predict the risk for AE. The nomogram demonstrated good predictive ability for AE using four common patient variables collected on admission; more specifically, age, C. protein, C. chloride, and spontaneous remission. To the best of our knowledge, this is the first study to report a clinically applicable predictive model for the early estimation of AE.

This study identified age as an independent risk factor for pediatric AE (OR = 1.44, 95% CI: 1.09–1.91). An article published in 2024 indicates that the incidence of overall AE in children ≤ 18 years, estimated at 1.54 per million children per year in the Netherlands, age of onset of AE ranged from 8 to 16 years for AE overall, whereas age of onset for NMDAR-AE ranged from 3.5–17 years in the identified studies (Santoro et al., 2024). Studies have shown that age is related to the prognosis of autoimmune encephalitis (Broadley et al., 2019; Pretkalnina et al., 2024). There are significant differences in the causes (antibody types) of AE among children of different age groups (Hardy, 2022), such as anti-NMDA-receptor encephalitis largely affects young people (Dalmau et al., 2008). Furthermore, age also affects the manifestation of clinical symptoms in autoimmune encephalitis and the response to immunotherapy (Armangue et al., 2018). Patients that were 4 years old or younger developed a syndrome punctuated by choreoathetosis, decreased level of consciousness and frequent seizures or infantile spasms, whereas older children and adults developed predominant change of behavior and psychiatric symptoms sometimes accompanied by seizures, in addition, older children and adults were more likely to respond to immunotherapy (Armangue et al., 2018). Previous studies were all consistent with our research.

In this study, our data demonstrated that C. protein was the important risk factor for AE. Elevated CSF protein can be used as a specific marker of CNS injury, the increase of CSF protein reflects the abnormal immune inflammatory response in central nervous system (Xu et al., 2021; Yan et al., 2024). Studies have shown that in the cerebrospinal fluid analysis of patients with autoimmune encephalitis, normal or mildly increased protein concentration (Dalmau et al., 2011; Cellucci et al., 2020; Zrzavy et al., 2021). The “Expert Consensus on the Diagnosis and Treatment of Autoimmune Encephalitis in China (2017)” also points out that routine cerebrospinal fluid biochemical tests may yield normal results or show mild inflammatory changes, namely mild lymphocytosis and/or a slight increase in protein levels. The mechanism by which proteins increase in the cerebrospinal fluid of patients with autoimmune encephalitis maybe as follows: (1) Inflammation of the central nervous system (CNS) increases the permeability of the blood-brain barrier, causing large molecular proteins (such as albumin) in the plasma to passively leak into the cerebrospinal fluid (Tumani et al., 2009). (2) Intrathecal immunoglobulin synthesis, such as oligoclonal bands (Graus et al., 2016; Deisenhammer et al., 2019). Research has confirmed the presence of specific autoantibodies produced by intrathecal plasma cells in the CSF of patients with anti - N - methyl-D-aspartate receptor (NMDAR) encephalitis (Gresa-Arribas et al., 2014). (3) Protein release within cells after neuronal damage, such as total Tau protein, neurofilament light chain (NFL), and other proteins, leading to an increase in the total protein level in cerebrospinal fluid (Mariotto et al., 2019). High level of CSF protein content predicts poor prognosis of AE patients (Yan et al., 2024). A study published in 2024 indicates that non - AE is primarily infectious encephalitis, and the C. protein of AE is significantly lower than that of infectious encephalitis (Moser et al., 2024). This is consistent with the fact that the protein levels in the AE group in this study were significantly lower than those in the non-AE group.

Our model incorporates new predictive factors: C. chloride. A study from China shows that the comparison of CSF chloride content, all patients had high chloride content, the chloride content of anti-LGI1 group was lower than that of anti-NMDAR group (*P* < 0.05),furthermore, elevated CSF chloride content were highly correlated with ICU admission(*P* = 0.006) (Yan et al., 2024). The mechanism of increased chloride ions remains unclear. It may be related to the dysfunction of the blood-cerebrospinal fluid barrier caused by inflammation or the imbalance of local ion homeostasis. This remains an area that requires further in-depth research in the future.

The AE patient’s neurological symptoms can achieve spontaneous remission after an episode (OR = 21.14, 95% CI:3.17–141.17). Patients slowly wake from coma as their autonomic functions stabilize, respiration recovers, and dyskinesias subside; During this period patients can become psychotic and agitated again, calming as they recover further (Dalmau et al., 2011). Orofacial dyskinesias were the most common; these included grimacing, masticatory-like movements, and forceful jaw opening and closing, resulting in lip and tongue injuries or broken teeth, progressing to a catatonic-like state, with periods of akinesis alternating with agitation, and diminished or paradoxical responses to stimuli (Dalmau et al., 2008). Case reports indicate that patients with AE may experience spontaneous remission (Najjar et al., 2011; Al-Diwani et al., 2012; Ramanan et al., 2018). There is also a case report of a patient with anti-VGKC antibody encephalitis. Although the clinical symptoms (encephalopathy) persisted for a long time and did not fully recover, the researchers observed that, without specific intervention, the inflammatory-related abnormal changes on the patient’s brain MRI spontaneously disappeared, and at the same time, the antibody titer in the circulation also decreased. This provides imaging and serological evidence for “spontaneous remission” (Novoselova et al., 2020). These findings collectively confirm that the spontaneous remission of patients following the onset of neuropsychiatric symptoms serves as an independent risk factor for autoimmune encephalitis. Symptom fluctuations or early improvements in some AE patients should not serve as a basis for delaying the initiation of immunotherapy (Al-Diwani et al., 2012). Delayed treatment may lead to permanent neuropsychiatric sequelae. Early identification and timely intervention remain crucial for improving the prognosis.

The use of a nomogram for estimating the risk for AE in patients is a new concept. Our predictive model is visual and easy to use, enabling early and accurate identification of patients with AE who require immunotherapy. By calculating the total points based on four easily accessible clinical variables (age, C. protein, C. chloride, and spontaneous remission), physicians can predict the risk for AE. The predictive model demonstrated excellent discrimination, with an AUC of 0.976. In addition, the calibration curves were well fitted. By using this predictive model, early and accurate identification of patients who are likely to require AE will enable closer monitoring for optimize allocation of medical resources, which can facilitate clinical decision-making and may potentially improve the clinical outcomes of patients with AE.

This study has several limitations. First, the sample size was relatively limited (n = 88, with 37 events), and the Events Per Variable (EPV = 9.2) was marginally below the recommended threshold of EPV PV ed (n = 88, with 37 events), and the Events Per Varent estimation. Second, clinical data from patients were retrospectively collected; thus, inherent bias was unavoidable, variable selection involved a multi-step process (differential screening, LASSO regression, univariable logistic regression, and multivariable logistic regression). The Bootstrap internal validation was applied only to the final model structure and therefore may not fully capture the optimism introduced during the variable selection procedure itself, this is likely a key reason why the calibration slope (0.777) deviated from the ideal value of 1.0 (**Supplementary Table 1**). Third, this study was a single-center retrospective analysis without external validation; the generalizability of the model remains to be confirmed in independent cohorts. Nevertheless, we believe that this visual predictive model represents a useful tool for clinical physicians who are focused on AE, but needs confirmation in future clinical trials.

## 5 Conclusion

This study identifies critical predictors and a high-performance predictive model for pediatric AE. We developed a clinically applicable nomogram for predicting the risk for AE. This nomogram enables early and accurate estimation of AE risk when patients are admitted to the hospital, which may provide guidance for clinical decision-making. These findings enable targeted risk stratification and early intervention, addressing a critical gap in pediatric AE management.

## Data Availability

The raw data supporting the conclusions of this article will be made available by the authors, without undue reservation.
