# Supplementary material for: Predictors for identifying autoimmune encephalitis in pediatric patients
Source: Front Cell Infect Microbiol. 2026 Jul 2;16:1827367. doi: 10.3389/fcimb.2026.1827367 (PMC13372578; doi:10.3389/fcimb.2026.1827367)
Supplement: Supplementary file 1 [file Table1.doc]

Table 1. Results of Bootstrap internal validation

| **Metric** | **Apparent** | **Bootstrap-corrected** | **Optimism** |
| --- | --- | --- | --- |
| AUC (C-statistic) | 0.976 | 0.968 | 0.007 |
| Calibration Slope | 1.000 | 0.777 | 0.223 |
| Calibration Intercept | 0.000 | 0.006 | — |
| Mean Absolute Error | — | 0.012 | — |
